# Performance evaluation of Novaplex SARS-CoV-2 variants assay kit series for SARS-CoV-2 detection using single nucleotide polymorphisms

**DOI:** 10.1099/acmi.0.000447

**Published:** 2022-10-27

**Authors:** Shinji Ogihara, Kotaro Aoki, Mami Nagashima, Kenji Sadamasu, Yoshikazu Ishii, Kazuhiro Tateda

**Affiliations:** ^1^​ Department of Collaborative Regional Infection Control, Toho University School of Medicine, 5-21-16 Omori-nishi, Ota-ku, Tokyo 143-8540, Japan; ^2^​ Department of Microbiology and Infectious Diseases, Toho University School of Medicine, 5-21-16 Omori-nishi, Ota-ku, Tokyo 143-8540, Japan; ^3^​ Department of Microbiology, Tokyo Metropolitan Institute of Public Health, 3-24-1 Hyakunin-cho, Shinjuku-ku, Tokyo 169-0073, Japan

**Keywords:** limit of detection, Novaplex SARS-CoV-2 variants assay kit series, SARS-CoV-2, single nucleotide polymorphism, validation, variant

## Abstract

Severe acute respiratory syndrome coronavirus 2 (SARS-CoV-2) variants have received increasing attention globally because of their increased transmissibility and potential to escape immunity. Although whole-genome sequencing is the gold standard method for SARS-CoV-2 mutation detection and lineage determination, it is costly and time-consuming. However, SARS-CoV-2 variants can be identified based on select variant-specific single nucleotide polymorphisms (SNPs) in the spike protein-encoding gene (*S*). This study validated and compared the limit of detection (LOD) of L452R, N501Y, HV69/70 del and E484K as variant-specific SNPs of the *S* gene and *RdRP* as a SARS-CoV-2-specific gene, using the Novaplex SARS-CoV-2 variants assay kit series. For three SARS-CoV-2 lineages (B.1.617.2, B.1.1.7 and R.1), one strain per lineage was used. Variant-specific SNPs of the *S* gene were analysed using the Novaplex SARS-CoV-2 variants I assay and Novaplex SARS-CoV-2 variants II assay kits. Validation confirmed the LODs of the variant kits. The LOD for each target variant-specific SNP and *RdRP* was five RNA copies per reaction. The Novaplex SARS-CoV-2 variants assay kit series performs well and the LOD for SARS-CoV-2 detection and variant-specific SNP detection are consistent. The kits are suitable for use as routine laboratory tests for SARS-CoV-2 and variant-specific SNP detection in a single step, saving time and labour.

## Data summary

All supporting data, code and protocols have been provided within the article.

## Introduction

It is important to identify prevalent variants of severe acute respiratory syndrome coronavirus 2 (SARS-CoV-2) lineages to understand their epidemiology and effects on public health. As SARS-CoV-2 variants have increased infectivity and greater potential for immune escape than the wild-type, their trends are being monitored globally [1, 2], and the analysis of SARS-CoV-2 lineages should continue in the future. In November 2021, a new variant strain, B.1.1.529, was reported, and concerns have been raised about the increased infectivity and transmissibility of this strain [3]. Although whole-genome sequencing (WGS) is essential for analysing SARS-CoV-2 lineages, it is difficult to perform on a wide scale because it is costly and time-consuming. WGS of SARS-CoV-2 variants of concern (VOCs) allows the strains to be inferred by detecting certain variant-specific single nucleotide polymorphisms (SNPs) in the spike protein-encoding gene (*S*). Commercial tests that use these variant-specific SNPs as markers for reverse-transcription quantitative PCR (RT-qPCR)-based detection have been developed [4, 5].

Many studies have evaluated the limit of detection (LOD) of commercial assays for SARS-CoV-2 detection [6, 7]. However, only a few studies have evaluated commercial assay kits for detecting variant-specific SNPs of SARS-CoV-2. Due to their rapid development, the performance of commercial variant assay kits has not been adequately evaluated. Previous reports have shown the LOD of N501Y, HV69/70 del and E484K [4, 5]. However, the LOD of each variant-specific SNP and the SARS-CoV-2-specific gene were different, and the LOD of the variant-specific SNPs was higher than the LOD of the SARS-CoV-2-specific genes such as *ORF1a/b*, *E*, *N* and *S*. Furthermore, there are no reports on the LOD of L452R of the widespread B.1.617.2 lineage. Novaplex SARS-CoV-2 variants I assay and Novaplex SARS-CoV-2 variants II assay kits are multiplex PCR kits for detecting seven variant-specific SNPs, including L452R, and SARS-CoV-2-specific genes [7].

## Objectives

This study aimed to validate and compare the LOD of L452R, N501Y, HV69/70 del and E484K as variant-specific SNPs in the *S* gene and *RdRP* as a SARS-CoV-2-specific gene, using the Novaplex SARS-CoV-2 variants assay kit series. In addition, the diagnostic accuracy for detecting VOCs was evaluated.

## Study design

Three isolated and cultured strains of SARS-CoV-2, each belonging to a different lineage, from the Tokyo Metropolitan Institute of Public Health (Japan) were used. The strains belonged to the B.1.617.2 (GISAID accession ID: EPI_ISL_2378732), B.1.1.7 (GISAID accession ID: EPI_ISL_1041952) and R.1 (GISAID accession ID: EPI_ISL_1041945) lineages, as determined by WGS [1].

Genomic RNA of cultured SARS-CoV-2 was extracted using the QIAamp viral RNA mini kit (QIAGEN); RNA was isolated at the Tokyo Metropolitan Institute of Public Health (Japan). RNA copy numbers of each SARS-CoV-2 lineage were determined using the PrimeScript II high fidelity RT-PCR kit (Takara Bio) for reverse-transcription PCR (RT-PCR), and the QuantStudio 3D digital PCR master mix v2 (Thermo Fisher Scientific) and 3D digital PCR system (Thermo Fisher Scientific) for digital PCR (dPCR) with N2 primers and probes developed by the National Institute of Infectious Disease in Japan [8]. Variant-specific SNPs in the *S* gene were analysed using the Novaplex SARS-CoV-2 variants I assay kit and Novaplex SARS-CoV-2 variants II assay kit (Seegene), according to the manufacturer’s instructions, using the CFX96 Touch real-time PCR detection system (Bio-Rad). The RNA template volume for RT-PCR, dPCR and RT-qPCR was 5 µl. RT-PCR, dPCR and RT-qPCR were performed at the Toho University School of Medicine (Tokyo, Japan). The LODs of the following variant-specific SNPs were verified: L452R in the B.1.617.2 lineage; N501Y, HV69/70 del and *RdRP* in the B.1.1.7 lineage; and E484K in the R.1 lineage.

The test results and cycle threshold (*C*_t_) values were automatically determined by the Seegene Viewer v1.0 software (Seegene). The *C*_t_ value was set to the indicated value +3 because the indicated value did not include the first three cycles.

Validation confirmed the LODs of the variant kits. Samples were adjusted to 1000 copies μl^−1^ according to the N2 RNA copy number of each lineage. For the LOD, samples with three different dilutions (10, 5 and 2.5 RNA copies per reaction) were prepared using each lineage at 1000 copies μl^−1^ and diethylpyrocarbonate (DEPC)-treated water. The LOD was determined for each target gene in 20 replicates of samples with a particular RNA copies per reaction level using hit rate analysis for each replicate (the minimum concentration yielding at least 95 % positive results) and reported as an RNA copies per reaction value.

## Results

The LOD for each target variant-specific SNP and *RdRP* was five RNA copies per reaction (Table 1). The mean *C*_t_ values±sd of 20 replicates of testing for the LOD in samples with five RNA copies per reaction were as follows: 37.50±1.07 for L452R in the B.1.617.2 lineage, 38.65±0.58 for N501Y in the B.1.1.7 lineage, 32.66±0.79 for HV69/70 del in the B.1.1.7 lineage, 36.21±0.71 for *RdRP* in the B.1.1.7 lineage, and 38.51±0.83 for E484K in the R.1 lineage.

**Table 1.** LODs of the Novaplex SARS-CoV-2 variants assay series for detecting SARS-CoV-2 SNPs and the SARS-CoV-2 *RdRP* gene

No. of copies per reaction	SARS-CoV-2 SNP									
	L452R		N501Y		HV69/70 del		E484K		*RdRP*	
	Positive rate	Mean *C*_t_ value (sd)	Positive rate	Mean *C*_t_ value (sd)	Positive rate	Mean *C*_t_ value (sd)	Positive rate	Mean *C*_t_ value (sd)	Positive rate	Mean *C*_t_ value (sd)
10	100 % (20/20)	36.38 (0.65)	100 % (20/20)	38.16 (0.65)	100 % (20/20)	34.79 (0.65)	100 % (20/20)	37.23 (0.49)	100 % (20/20)	35.19 (0.50)
5	95 % (19/20)	37.50 (1.07)	100 % (20/20)	38.65 (0.58)	100 % (20/20)	32.66 (0.79)	100 % (20/20)	38.51 (0.83)	95 % (19/20)	36.21 (0.71)
2.5	80 % (8/10)	38.01 (0.70)	85 % (17/20)	39.97 (0.86)	90 % (18/20)	36.66 (0.65)	85 % (17/20)	39.71 (0.75)	90 % (18/20)	37.41 (0.77)

## Discussion

We expected the LOD of each target gene to be equal. If the SARS-CoV-2-specific gene is negative, the variant-specific SNPs are also negative. However, if the LOD of each target is different, it is difficult to judge whether it is undetectable or a wild-type. Furthermore, in previous studies, the LOD of the N501Y SNP has been reported to be 10-fold higher than that of the SARS-CoV-2-specific genes *ORF1a/b*, *N* and *S* [4, 5]. In this study, the *C*_t_ values of the LOD for each target gene varied from 32.66 to 38.65; however, the LOD for all target genes was five RNA copies per reaction. These assays automatically changed the threshold position of each target gene, so we considered that the LOD of each target gene showed the same value, despite the differences in the *C*_t_ value of each gene; thus, making it easy to identify undetected, wild-type and variant-specific SNPs in variant-specific SNP analysis.Variant-specific SNP detection is generally performed after SARS-CoV-2 detection, which is laborious and time-consuming. However, if the LOD of variant-specific SNPs and SARS-CoV-2 detection is the same as that of SARS-CoV-2 detection, as in the kits, then the analysis can be performed in one step, saving time and effort.

In this study, we were unable to validate the assay for detecting SARS-CoV-2 B.1.529 and the K417T, K417N and W152C SNPs in the kit, because we did not have any samples of SARS-CoV-2 RNA with these variant-specific SNPs. Furthermore, a low LOD was determined in this study. This may be related to the fact that the samples used were cultures with few impurities. Clinical samples are expected to contain PCR inhibitors; hence, the LOD may be higher for clinical samples. However, we did not validate any patient specimen-derived samples.

The Novaplex SARS-CoV-2 variants assay kit series had low LODs and the same LOD values for the L452R, N501Y, HV69/70 del and E484K SNPs, and the *RdRP* gene of SARS-CoV-2. The Novaplex SARS-CoV-2 variants assay kit series is superior to other detection systems or kits because the LOD for SARS-CoV-2 detection and variant-specific SNP detection are consistent. The current dominant SARS-CoV-2 variant (B.1.617) will possibly be replaced by B.1.529 [9]. Conducting surveillance using the Novaplex SARS-CoV-2 variants assay kit series could provide a rapid and accurate understanding of the epidemiology and public-health implications.
